# Verification of QTL for Grain Starch Content and Its Genetic Correlation with Oil Content Using Two Connected RIL Populations in High-Oil Maize

**DOI:** 10.1371/journal.pone.0053770

**Published:** 2013-01-08

**Authors:** Guohu Yang, Yongbin Dong, Yuling Li, Qilei Wang, Qingling Shi, Qiang Zhou

**Affiliations:** College of Agriculture, Henan Agricultural University, Key Laboratory of Physiological Ecology and Genetic Improvement of Food Crops in Henan Province, Zhengzhou, People’s Republic of China; New Mexico State University, United States of America

## Abstract

Grain oil content is negatively correlated with starch content in maize in general. In this study, 282 and 263 recombinant inbred lines (RIL) developed from two crosses between one high-oil maize inbred and two normal dent maize inbreds were evaluated for grain starch content and its correlation with oil content under four environments. Single-trait QTL for starch content in single-population and joint-population analysis, and multiple-trait QTL for both starch and oil content were detected, and compared with the result obtained in the two related F_2∶3_ populations. Totally, 20 single-population QTL for grain starch content were detected. No QTL was simultaneously detected across all ten cases. QTL at bins 5.03 and 9.03 were all detected in both populations and in 4 and 5 cases, respectively. Only 2 of the 16 joint-population QTL had significant effects in both populations. Three single-population QTL and 8 joint-population QTL at bins 1.03, 1.04–1.05, 3.05, 8.04–8.05, 9.03, and 9.05 could be considered as fine-mapped. Common QTL across F_2∶3_ and RIL generations were observed at bins 5.04, 8.04 and 8.05 in population 1 (Pop.1), and at bin 5.03 in population 2 (Pop.2). QTL at bins 3.02–3.03, 3.05, 8.04–8.05 and 9.03 should be focused in high-starch maize breeding. In multiple-trait QTL analysis, 17 starch-oil QTL were detected, 10 in Pop.1 and 7 in Pop.2. And 22 single-trait QTL failed to show significance in multiple-trait analysis, 13 QTL for starch content and 9 QTL for oil content. However, QTL at bins 1.03, 6.03–6.04 and 8.03–8.04 might increase grain starch content and/or grain oil content without reduction in another trait. Further research should be conducted to validate the effect of these QTL in the simultaneous improvement of grain starch and oil content in maize.

## Introduction

Maize is widely used as an important renewable resource for industrial materials, biodiesel production, and dietary consumption by humans and animals all over the world [1], [2], [3], [4]. Since starch is rich in caloric and oil is high in energy content, both starch and oil content in maize grain have been improved for a long time [5]. After Hopkins (1899) initiated the selection of high and low grain oil content in maize [6], several research went along with the improvement of grain chemical composition using different genetic backgrounds [5], [7], [8], [9], [10]. On the whole, two major practical objectives in maize breeding and genetic research are how to improve grain quality for normal maize and how to improve grain yield for quality maize.

Several early research demonstrated that the negative correlation between grain starch and oil content was resulted in the reduction of starch content and grain yield along with the increase of oil content [4], [11], [12], [13]. Wassom et al. [9] found that starch content was positively correlated with kernel mass in BC_1_S_1_s (r_p_ = 0.67^**^) and with yield in TCs (r_p_ = 0.59^**^), while oil content was negatively correlated with kernel mass and starch content in BC_1_S_1_s (r_p_ = –0.29^**^, –0.75^**^) and with yield in TCs (r_p_ = –0.30^**^, –0.66^**^) by analyzing kernel traits in a backcross and testcross populations derived from Illinois High Oil (IHO) and the recurrent parent B73. Further negative correlations between starch and oil content at different levels were reflected in populations for QTL mapping using different genetic backgrounds, such as Illinois High Protein (IHP) and Illinois Low Protein (ILP) [5], [14], Illinois High Oil (IHO) and Illinois Low Oil (ILO) [9], [15], [16], [17], Beijing high-oil (BHO) [18], [19], tropical [8], Alexho Single-Kernel (ASK) high-oil [20], [21], and popcorn [13]. Great differences in QTL numbers, locations and effects for the same trait were also observed among those studies. Till now, only one high-oil QTL (*qHO6*) was applied to marker based selection, refined and cloned [20]. And the increase in seed oil with no change in seed weight was observed in its transgenic lines. Of course, the molecular basis of QTL for oil and starch content and their relationship should be extensively revealed in further research.

In our previous study, two connected F_2∶3_ populations have been used to identify QTL for grain oil and starch content and their associations [10]. Two connected RIL populations developed from the same two crosses as the F_2∶3_ populations have been derived and used to detect QTL for ear kernel traits and grain oil content [22]. In this study, the two connected RIL populations were used to detect and compare single-trait QTL for grain starch content in single-population and joint-population analysis, and multiple-trait QTL for grain starch and oil content under 4 environments as in the F_2∶3_ generations. Our first objective was to analyze the influence of genetic backgrounds, environments and generations on QTL detection for grain starch content. The second objective was to further reveal the genetic mechanism in controlling grain starch content and its correlation with oil content in high-oil maize. The third objective was to find consistent QTL in further research in QTL cloning, marker assisted breeding for starch improvement and the simultaneous improvement of starch and oil content in high-oil maize.

## Materials and Methods

### Population Development and Field Experiment

The development of the two RIL populations and the field experiment had been described in our previous research [22]. Briefly, one high-oil maize inbred GY220 was crossed with two normal corn inbreds 8984 and 8622 to make two connected RIL populations using single-seed descent method. GY220 was derived from the cycle 27 Alexander high-oil maize population and was selected and provided by China Agricultural University, which belonged to the Lancaster heterotic group [21], [23]. The two normal dent corn inbreds were developed in our laboratory and belonged to the Chinese Reid heterotic group. The RIL populations derived from 8984 × GY220 and 8622 × GY220 were 282 and 263 lines, and referred as population 1 (Pop.1) and population 2 (Pop.2), respectively.

The two RIL populations, along with their two respective parent lines, were planted in two adjacent trails under four environments, at three locations (Xuchang, Xinxiang, and Zhengzhou) in summer sowings (12 June) in Henan, and at Yinchuan in spring sowing (15 April), in China, in 2009. The α-design was used with one-row plots and two replications. Each row was 4 m long with 0.67 m between rows. Plots were planted by hand at a density of 60,000 plants ha^−1^. Standard cultivation management practices were used at each location [22].

### Trait Evaluation

As described by Yang et al. [22], three plants were sib-pollinated within each plot by hand to avoid xenia effect. After maturity, ears of the three plants were harvested and naturally dried. Grain starch and oil content were measured on grain samples mixed within each plot with a MATRIX-1 NIR Spectroscope (Bruker, Corporation, Germany) according to the method described by Dudley and Lambert [24].

### Phenotypic Data Analysis

Using the statistical software package SPSS 12.0, combined analysis of variance and correlation coefficients between starch and oil content were carried out following the standard procedures of a mixed model with a random genetic effect, and fixed environment and replicate effects according to the Henderson III method [25]. Since the genotype × environment interactions were significant for grain starch content in the two RIL populations, data under each environment were analyzed individually in further analysis. For comparison, combined analysis was conducted using simple average phenotypic data across four environments as in our previous articles [22]. Broad-sense heritabilities (H^2^_B_) for the two connected RIL populations on an entry mean basis were calculated by dividing the genotypic variance by the phenotypic variance [26]. Confidence intervals on heritability estimates were determined according to Knapp et al. [27].

### QTL Analysis within Single Population

The integrated genetic map for the two populations included 313 SSR markers, and was 2349.4 cM long with an average interval of 7.50 cM [22]. It was obtained through BioMercator 2.1 software [28].

The composite interval mapping (CIM) [29] with Model 6 of the Zmapqtl procedure in QTL Cartographer Version 2.5 was used in QTL mapping for grain starch content under each environment and combined across four environments in both populations. Thresholds for logarithm of odds (LOD) scores to identify QTL were estimated by permutation tests with a minimum of 1,000 replicates [30]. QTL positions were assigned to relevant regions at the point of a maximum LOD score. Confidence intervals were calculated by subtracting one LOD unit on each side of the maximum LOD position [31]. Multiple interval mapping (MIM) in QTL Cartographer Version 2.5 was used to analyze interactions between detected QTL for grain starch content [32], [33]. Significant thresholds were identified by the quick method for computing approximate thresholds for QTL detection [34].

To further test the genetic relationships between grain oil and starch content, a multiple-trait version of the composite interval mapping was used detect multiple-trait QTL [35] with QTL Cartographer Version 2.5 [33]. A significance threshold was also identified by the quick method for computing approximate thresholds for quantitative trait loci detection [34].

### Joint-population QTL Analysis for the Two Population

To combine the two populations, a joint-population analysis was performed using the joint inclusive composite interval mapping (JICIM) method [36]. QTL mapping for the nested association mapping (NAM) design in QTL IciMapping Version 3.2 (available from www.isbreeding.net) was used to detect joint-population QTL for grain starch content under each environment and combined across four environments. Empirical LOD score of 2.5 were used to declare the existence of QTL. The additive effects, positions, LOD values, and the phenotypic variance explained (R^2^) for each detected QTL were obtained.

For each detected QTL, positive and negative additive effects indicated that the allele from the high-oil parent GY220 and the dent corn inbred lines 8984 or 8622 increased the value of the trait, respectively. QTL were named according to “q”+“environment abbreviation”+“trait abbreviation”+“population number”+“–”+“chromosome number”+“–”+“QTL number”. For joint-population QTL, the “population number” was omitted. Multiple-trait QTL for the two traits were named according to “q”+“environment abbreviation”+“abbreviation for the two traits (starch and oil, SO)”+“population number”+“–”+“chromosome number”+“–”+“QTL number”. For example, the front words “qc”, “qx”, “qz”, “qy” and “q” represented QTL detected at Xuchang, Xinxiang, Zhengzhou, Yinchuan and in combined analysis, respectively.

## Results

### Combined Variance, Heritability and Performance for Grain Starch Content in the Two Connected RIL Populations

The result of combined analysis of variance showed that all variance components were significant for grain starch content in both populations except environment variance (σ_E_^2^) in Pop.1 (Table 1). The broad sense heritability (h_B_^2^) estimates were a little higher in Pop.1 (0.72) than in Pop.2 (0.69). Among the three parent lines, the value for grain starch content for the high-oil maize inbred GY220 was lower than that of the two normal inbreds 8984/8622 under all environments. But the difference between the two normal inbreds was not obvious (Table 1). In the two populations, all values of grain starch content showed normal distribution with a wide range of variation and transgressive segregation exceeding both parent values. The variance coefficients (CV%) were higher in Pop.2 than in Pop.1 under all environments.

**Table 1:** Combined variance analysis, heritability and phenotypic performance of grain starch content for the two RIL populations under each environment.

Population	Environment	Parent		RIL population					Variance component			Heritability	
		GY220	8984/8622 a	Range	Mean±SD	CV%	Skewness	Kurtosis	σ_G_^2^	σ_E_^2^	σ_GE_^2^	h_B_^2^	C.I. on h_B_^2^ b
Pop.1	Xuchang	60.84	66.25	59.45–71.06	65.06±1.85	2.84	−0.09	0.21	4.13^**^	5.13	1.15*	0.72	0.65–0.78
	Xinxiang	61.58	66.80	59.51–69.73	65.42±1.68	2.57	−0.24	0.04					
	Zhengzhou	62.81	64.01	60.54–69.77	65.40±1.63	2.49	−0.32	0.18					
	Yinchuan	62.35	66.97	62.02–69.52	65.65±1.43	2.18	0.11	−0.15					
	Combined	61.90	66.01	59.72–69.91	65.37±1.29	1.97	−0.29	1.04					
Pop.2	Xuchang	60.84	65.75	58.79–70.87	65.25±1.96	3.01	−0.12	0.41	3.75^**^	6.14*	1.24^**^	0.69	0.58–0.74
	Xinxiang	61.58	67.45	60.11–72.49	66.01±1.86	2.82	−0.09	0.41					
	Zhengzhou	62.81	66.88	58.61–70.28	65.52±1.82	2.78	−0.46	0.72					
	Yinchuan	62.35	67.40	61.29–71.99	65.86±1.86	2.82	0.06	0.44					
	Combined	61.90	66.87	59.78–71.77	65.64±1.37	2.09	0.07	0.22					

* Significant at P<0.05, ^**^ Significant at P<0.01.

a ‘8984’ was the parent in Pop.1, and ‘8622’ was the parent in Pop.2.

b *C.I.* confidence interval.

Significantly negative phenotypic and genotypic correlations were observed between grain starch and oil content under four environments and in combined analysis in both connected populations, −0.21^**^–−0.35^**^ in Pop.1, and −0.26^**^–−0.40^**^ in Pop.2 (Table 2).

**Table 2:** Phenotypic and genotypic correlations between grain starch and oil content for the two RIL populations under each environment and in combined analysis.

Population	Environment	Genotypic correlation	Phenotypic correlation
Pop.1	Xuchang	−0.22**	−0.21**
	Xinxiang	−0.35**	−0.33**
	Zhengzhou	−0.23**	−0.22**
	Yinchuan	−0.24**	−0.22**
	Combined	−0.24**	−0.23**
Pop.2	Xuchang	−0.27**	−0.26**
	Xinxiang	−0.29**	−0.28**
	Zhengzhou	−0.28**	−0.27**
	Yinchuan	−0.29**	−0.28**
	Combined	−0.40**	−0.39**

** Significant at P<0.01.

### QTL Analysis for Grain Starch Content within Single Population

**Figure 1:**
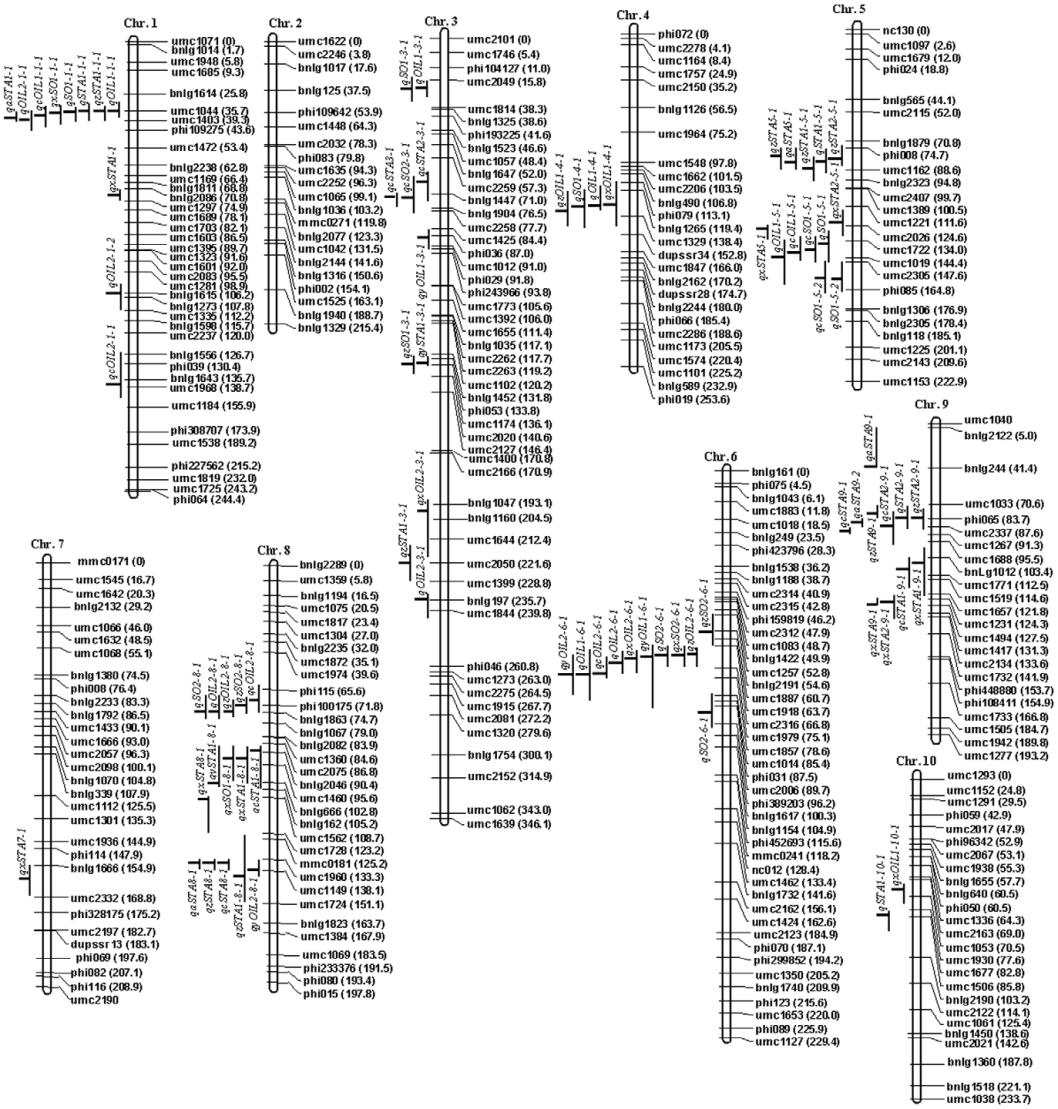
The integrated genetic map, single-trait QTL for grain starch content in single-population and joint-population analysis, and multiple-trait QTL for starch and oil content on chromosomes. QTL are showed to the left of chromosome. One-LOD support intervals are indicated by *vertical bars*, and the maximum LOD peak positions are indicated by *horizontal bars*.

Since the variances of genotype × environment interaction (σ_GE_^2^) for grain starch content were significant in both populations, QTL mapping was conducted under each environment. For comparison, combined analyses using means across the four environments were also conducted. A total of 20 single-population QTL were detected under four environments and in combined analyses in the two populations (Table 3, Fig. 1), 12 in Pop.1 and 8 in Pop.2. Except no QTL were detected for Pop.2 at Yinchuan, 1–4 QTL were detected in each case.

**Table 3:** QTL detected for grain starch content in the two RIL populations under each environment and in combined analysis.

Population	Environment	QTL	Flanking marker	Interval (cM) a	Bin locus^b^	Position^c^	LOD	A^d^	R^2^%^e^
Pop.1	Xuchang	qcSTA1-8-1	umc2075-bnlg2046	12.4 (12.7/0)	8.03–8.04	110.4	2.94	0.34	4.47
		qcSTA1-9-1	umc1688-umc1771	12.8 (10.0/2.8)	9.03–9.04	64.0	2.5	−0.34	4.56
	Xinxiang	qxSTA1-8-1	bnlg2046-umc1562	3.9 (2.0/1.9)	8.04–8.05	112.4	2.95	−0.32	4.61
		qxSTA1-9-1	umc1688-umc1771	12.8 (8.0/4.8)	9.03–9.04	62.0	2.98	−0.36	5.91
	Zhengzhou	qzSTA1-1-1	umc1044-phi109275	14.9 (0/14.9)	1.03	51.1	2.5	0.31	4.03
		qzSTA1-3-1	umc1399-umc1844	11.1 (0/11.1)	3.07–3.08	245.4	2.76	−0.32	3.98
		qzSTA1-5-1	bnlg1879-umc1162	17.8 (17.8/0)	5.02–5.04	75.3	5.31	−0.47	8.03
		qzSTA1-8-1	umc1149-umc1960	8.1 (6.1/2.0)	8.06	159.9	2.93	−0.32	4.81
	Yinchuan	qySTA1-3-1	phi053-umc1174	2.4 (2.0/0.4)	3.05	189.5	2.53	−0.32	4.01
		qySTA1-8-1	bnlg2046-umc1562	3.9 (3.9/0)	8.04–8.05	114.3	2.56	−0.33	4.35
	Combined	qSTA1-1-1	umc1044-phi109275	14.9 (0/14.9)	1.03	51.1	3.07	0.26	5.46
		qSTA1-5-1	bnlg1879-umc1162	17.8 (12.0/5.8)	5.02–5.04	69.5	3.15	−0.33	3.24
Pop.2	Xuchang	qcSTA2-3-1	umc2259-bnlg1447	13.7 (8.0/5.7)	3.02–3.03	65.2	5.74	0.61	12.35
		qcSTA2-9-1	phi065-umc1267	7.6 (4.0/3.6)	9.03	87.7	3.36	−0.42	5.97
	Xinxiang	qxSTA2-5-1	umc1389-umc1722	33.5 (22.0/11.5)	5.03–5.05	122.5	2.61	−0.46	7.55
		qxSTA2-9-1	umc1657-umc1494	5.7 (4.0/1.7)	9.05	125.8	3.99	−0.47	7.24
	Zhengzhou	qzSTA2-5-1	phi008-umc1389	25.8 (6.0/19.8)	5.03	80.7	2.53	−0.43	7.32
		qzSTA2-9-1	phi065-umc1267	7.6 (4.0/3.6)	9.03	83.7	2.58	−0.41	5.43
	Combined	qSTA2-9-1	phi065-umc1267	7.6 (4.0/3.6)	9.03	83.7	3.35	−0.32	6.95
		qSTA2-10-1	umc1506-bnlg2190	17.4 (0/17.4)	10.05–10.06	85.8	3.48	0.32	5.69

a Values in the brackets were the intervals between QTL and its ﬂanking markers.

b Bin locations of the ﬂanking markers from Maize GDB (http://www.maizegdb.org).

c Genetic map position by cM.

d A means additive effects estimated with QTL Cartographer.

e R^2^ means percent of phenotypic variance explained by each QTL.

These QTL were located on chromosomes 1, 3, 5, 8, 9 and 10. The QTL on chromosomes 3, 5 and 9 were all detected in both populations. The QTL on chromosome 9 was located at the same or near bins 9.03–9.04 in 6 cases, 2 cases in Pop.1 and 4 cases in Pop.2. The QTL on chromosome 5 at bins 5.02–5.04/5.03–5.05 were detected in 2 cases for both populations. The QTL on chromosome 3 were detected in 3 cases, 2 cases in Pop.1 and 1 case in Pop.2. But they were located at different bins, at bins 3.07–3.08/3.05 in Pop.1 and at bins 3.02–3.03 in Pop.2. The QTL on chromosomes 1 and 8 were only detected in Pop.1 in 2 and 4 cases, respectively. One QTL on chromosome 10 at bins 10.05–10.06 was detected in Pop.2. Except five QTL at bins 1.03, 3.02–3.03, 8.03–8.04 and 10.05, the positive alleles of other 14 QTL were all contributed by the normal corn parents. The contribution to phenotypic variation for qcSTA2-3-1 was the largest (12.35%), which could be considered as a major QTL. But those for other 19 individual QTL were all smaller than 10% (between 3.24% and 8.03%), they were all minor QTL. The marker intervals of QTL positions were 2.4–33.55 cM, with an average of 12.59 cM. The QTL numbers with marker intervals <5 cM, 5–10 cM, 10–15 cM and >15 cM were 3, 5, 6 and 6, accounting for 15.0%, 25.0%, 30.0% and 30.0%, respectively. Three QTL (qxSTA1-8-1, qySTA1-3-1 and qySTA1-8-1) with intervals 3.9 cM, 2.4 cM and 3.9 cM could be considered as being fine-mapped.

Among detected QTL, 9 pairs of digenic interactions were identified under each environment and in combined analysis in the two populations (Table 4), which included 3 QTL×QTL (additive×additive effects), 4 QTL×genetic background (marker intervals on which none QTL were detected) and 1 genetic background×genetic background interactions. Those interactions were related with all the six chromosomes with QTL distributed. Besides, 5 marker intervals on chromosomes 4 and 7 were also included, on which QTL were failed to be detected. All the values of interaction effects were low, from 0.4%–4.3%. This result suggested that the contributions of digenic interactions to the performance of grain starch content were all minimal in both populations. Obvious influence of environments on epistasis could also be observed. For example, although both QTL on chromosomes 8 and 9 (at the same marker interval umc1688–umc1771) were detected at Xuchang and Xinxiang in Pop.1, different digenic interactions were detected, umc1847–bnlg2244 ×qcSTA1-9-1 at Xuchang vs. qxSTA1-8-1×qxSTA1-9-1 at Xinxiang.

**Table 4:** Digenic epistatic interactions among detected QTL for grain starch content under each environment and in combined analysis for the two RIL populations.

Population	Environment	QTL/Marker interval 1	QTL/Marker interval 2	LOD	Effect	R^2%^
Pop.1	Xuchang	umc1847-bnlg2244 (4.07–4.08)	qcSTA1-9-1 (9.03–9.04)	0.55	−0.17	1.1
	Xinxiang	qxSTA1-8-1 (8.04–8.05)	qxSTA1-9-1 (9.03–9.04)	2.11	0.29	4.3
	Zhengzhou	qzSTA1-8-1 (8.06)	umc1384-umc1069 (8.07–8.08)	0.56	0.18	0.9
	Yinchuan	qySTA1-3-1 (3.05)	qySTA1-8-1 (8.04–8.05)	0.20	0.09	0.4
	Combined	qSTA1-1-1 (1.03)	phi046-bnlg1754 (3.08–3.09)	1.18	−0.17	1.7
Pop.2	Xuchang	umc2286-umc1173 (4.09)	bnlg118-umc1225 (5.07–5.08)	1.59	−0.29	2.0
	Xinxiang	bnlg1666-umc2332 (7.04)	qxSTA2-9-1 (9.05)	0.94	−0.24	2.4
	Zhengzhou	bnlg589-phi019 (4.10–4.11)	phi328175-dupsr13 (7.04)	0.78	0.22	1.3
	Combined	qSTA2-9-1 (9.03)	qSTA2-10-1 (10.05–10.06)	0.66	0.13	1.1

### Joint-population QTL Analysis for Grain Starch Content Across the Two Connected RIL Populations

Using joint-population analysis across the two connected RIL populations by JICIM, 16 joint-population QTL for grain starch content were detected under each environment and in combined analysis, 3 at Xuchang, 5 at Xinxiang, 3 at Zhengzhou and 5 in combined analysis (Table 5, Fig. 1). No QTL was detected at Yinchuan. The contribution to phenotypic variation for an individual QTL varied between 11.39% and 23.45%, with 2.00%–95.82% in Pop.1 and 1.29%–26.33% in Pop.2. The marker intervals of QTL positions were 3.2–36.4 cM, with an average of 9.59 cM. The QTL numbers with marker intervals <5 cM, 5–10 cM, 10–15 cM and >15 cM were 8, 2, 5 and 1, accounting for 50.0%, 12.5%, 31.25% and 6.25%, respectively. Eight QTL (qcSTA8-1, qcSTA9-1, qxSTA1-1, qxSTA9-1, qzSTA8-1, qSTA1-1, qSTA8-1 and qSTA9-2) at bins 1.03, 1.04–1.05, 8.06, 9.03 and 9.05 with intervals 3.6 cM, 4.1 cM, 4.8 cM, 3.9 cM and 3.2 cM could be considered as being fine-mapped.

**Table 5:** QTL for grain starch content detected by joint-population analysis for the two population using JICIM.

Environment	QTL	Position a	LOD	R^2^(%)^b^	Left marker (Bin locus)^c^	Right marker (Bin locus)	Interval (cM)^d^	Additive genetic effectin each population		R^2^(%) in each population	
								Pop.1	Pop.2	Pop.1	Pop.2
Xuchang	qcSTA3-1	72.5	4.02	23.14	bnlg1447 (3.03)	bnlg1904 (3.04)	5.5 (1.5/4.0)	0.12	−0.87^*^	2.00	26.33
	qcSTA8-1	135.0	2.65	17.37	umc1960 (8.06)	umc1149 (8.06)	4.8 (1.7/3.1)	0.55^*^	−0.42	43.08	5.99
	qcSTA9-1	90.0	4.02	13.61	umc2337 (9.03)	umc1267 (9.03)	3.7 (2.4/1.3)	0.49^*^	0.70^*^	33.39	16.80
Xinxiang	qxSTA1-1	72.5	2.88	15.08	bnlg2086 (1.04)	umc1297 (1.05)	4.1 (1.8/2.3)	−0.59	0.25	48.30	2.19
	qxSTA5-1	127.5	4.12	18.39	umc2026 (5.05)	umc1722 (5.05)	9.4 (2.9/6.5)	0.53	0.79^*^	38.67	21.03
	qxSTA7-1	167.5	2.56	11.39	bnlg1666 (7.04)	umc2332 (7.04)	13.9 (12.6/1.3)	−0.21	−0.66	6.04	14.65
	qxSTA8-1	110.0	3.20	17.94	umc1562 (8.05)	umc1728 (8.06)	14.5 (1.3/13.2)	0.62^*^	−0.31	53.54	3.25
	qxSTA9-1	127.5	4.65	14.92	umc1231 (9.05)	umc1494 (9.05)	3.2 (3.2/0)	0.49	0.70^*^	33.68	16.69
Zhengzhou	qzSTA5-1	77.5	4.72	19.93	phi008 (5.03)	umc1162 (5.04)	13.9 (2.8/11.1)	0.83^*^	0.62^*^	95.82	12.82
	qzSTA8-1	135.0	2.81	16.76	umc1960 (8.06)	umc1149 (8.06)	4.8 (1.7/3.1)	0.62^*^	−0.30	54.48	3.13
	qzSTA9-1	80.0	3.10	12.92	umc1033 (9.02)	phi065 (9.03)	13.1 (9.4/3.7)	0.42	0.70^*^	25.05	16.48
Combined	qSTA1-1	37.5	2.52	13.28	umc1044 (1.03)	umc1403 (1.03)	3.6 (1.8/1.8)	−0.47^*^	0.15	30.81	1.29
	qSTA5-1	82.5	4.00	16.42	phi008 (5.03)	umc1162 (5.04)	13.9 (7.8/6.1)	0.61^*^	0.37	52.57	7.42
	qSTA8-1	135.0	3.36	21.61	umc1960 (8.06)	umc1149 (8.06)	4.8 (1.7/3.1)	0.51	−0.33	36.03	5.88
	qSTA9-1	40.0	3.18	23.45	bnlg2122 (9.01)	bnlg244 (9.02)	36.4 (35.0/1.4)	−0.41	0.46	24.05	11.87
	qSTA9-2	85.0	3.95	15.88	phi065 (9.03)	umc2337 (9.03)	3.9 (1.3/2.6)	0.33	0.61^*^	15.55	20.64

a Genetic map position by cM.

b R^2^ means percent of phenotypic variance explained by each QTL.

c Values in the brackets were the bin locations of the ﬂanking markers from Maize GDB (http://www.maizegdb.org).

d Values in the brackets were the intervals between QTL and its ﬂanking markers.

In comparison, 12 of 16 joint-population QTL were detected in the single-population analysis (Table 3). Although single-population QTL qSTA2-10-1 failed to be detected herein, 4 joint-population QTL (qxSTA1-1, qxSTA7-1, qSTA8-1 and qSTA9-1) could be considered as additional QTL detected in joint-population analysis. The LOD values and the contribution to phenotypic variations for most joint-population QTL were much higher than those in the single-population mapping, while their interval distances were much smaller. This reflected the higher mapping power of joint-population analysis. Ten of the 16 joint-population QTL with significant effects in only one population could be considered as rare QTL. However, 2 joint-population QTL (qcSTA9-1 and qzSTA5-1) had significant genetic effects in both populations.

### Multiple-trait QTL Analysis for Grain Starch Content with Grain Oil Content in the Two Connected RIL Populations

To further analyze the genetic correlations between starch and oil content, multiple-trait analysis for grain starch and oil content was conducted in both populations (Table 6; Fig. 1). Totally, 17 starch-oil QTL (qSO) were detected, including 3 at Xuchuang, 3 at Xinxiang, 3 at Zhengzhou and 8 in combined analysis. No starch-oil QTL were detected at Yinchuan in both populations. Ten and 7 starch-oil QTL were detected in Pop.1 and Pop.2, respectively. These QTL were located on chromosomes 1, 3, 4, 5 and 8 at bins 1.03, 3.05/3.01–3.03, 4.05–4.06, 5.05–5.06/5.06–5.07, 8.04–8.05, respectively.

**Table 6:** Multiple-trait QTL analysis of grain starch content with oil content in the two RIL populations under each environment and in combined analysis.

Population	Environment	QTL	Flanking marker	Interval (cM) a	Bin locus^b^	Position^c^	LOD
Pop.1	Xuchang	qcSO1-5-1	umc2026-umc1019	19.8 (14.0/5.8)	5.05–5.06	125.3	6.47
		qcSO1-5-2	umc2305-bnlg1306	30.0 (12.0/18.0)	5.06–5.07	147.5	6.47
	Xinxiang	qxSO1-1-1	umc1044-phi109275	14.9 (2.0/12.9)	1.03	53.1	4.1
		qxSO1-8-1	bnlg2046-umc1562	3.9 (2.0/1.9)	8.04–8.05	112.4	3.88
	Zhengzhou	qzSO1-3-1	phi053-umc1174	2.4 (2.4/0)	3.05	189.9	4.36
	Combined	qSO1-1-1	umc1044-phi109275	14.9 (0/14.9)	1.03	51.1	5.92
		qSO1-3-1	umc2049-bnlg1523	17.7 (4.0/13.7)	3.01–3.03	55.7	4.11
		qSO1-4-1	umc1548-umc1329	18.7 (12.0/6.7)	4.05–4.06	85.7	5.35
		qSO1-5-1	umc2026-umc1019	19.8 (12.2/7.6)	5.05–5.06	123.5	6.94
		qSO1-5-2	umc2305-bnlg1306	30.0 (10.0/20.0)	5.06–5.07	145.5	6.01
Pop.2	Xuchang	qcSO2-3-1	bnlg1447-bnlg1904	5.5 (2.0/3.5)	3.03–3.04	72.9	4.74
	Xinxiang	qxSO2-6-1	umc2316-umc1979	8.3 (4.0/4.3)	6.03–6.04	70.8	7.25
	Zhengzhou	qzSO2-6-1	umc2316-umc1979	8.3 (4.0/4.3)	6.03–6.04	60.7	4.83
		qzSO2-8-1	phi100175-bnlg1863	2.9 (0/2.9)	8.03	71.8	4.33
	Combined	qSO2-6-1	umc2316-umc1979	8.3 (4.0/4.3)	6.03–6.04	70.8	7.42
		qSO2-6-2	phi031-bnlg1617	12.8 (6.0/6.8)	6.04–6.05	93.5	5.81
		qSO2-8-1	phi100175-bnlg1863	2.9 (2.0/0.9)	8.03	73.8	4.72

a Values in the brackets were the intervals between QTL and its ﬂanking markers.

b Bin locations of the ﬂanking markers from Maize GDB (http://www.maizegdb.org).

c Genetic map position by cM.

In our previous research, 12 and 14 QTL for grain oil content were detected in the two populations, respectively [22]. They were located on chromosomes 1, 3, 4, 5, 6, 8 and 10, and related with 14 bin loci (1.03, 1.03–1.04, 1.05–1.06, 1.08, 3.01–3.03, 3.03–3.04, 3.06–3.08, 4.05–4.06, 5.05–5.06, 6.03–6.04, 6.04, 8.03, 8.06 and 10.04–10.05). Compared with single-trait QTL for both traits, no additional QTL was detected, which indicated QTL only detected in multiple -trait analysis. The graph of LOD curve peaks for grain starch and oil content changed simultaneously and in the same direction at 5 marker intervals in 8 cases, including phi053–umc1174 on chromosome 3 at Zhengzhou, umc1548–umc1329 on chromosome 4 in combined analysis in Pop.1, and umc2316–umc1979 on chromosome 6 at Xinxiang, at Zhengzhou and in combined analysis, phi100175–bnlg1863 on chromosome 8 at Zhengzhou and in combined analysis, and umc1601–bnlg1615 on chromosome 1 in combined analysis in Pop.2. These data suggested that there existed pleiotropic QTL controlling both traits simultaneously. Besides, the peaks of LOD curve graph for both traits changed in the same close direction at 4 marker intervals in 7 cases, including umc2026–umc1019 and umc2305–bnlg1306 on chromosome 5 at Xuchang and in combined analysis, and umc1044–phi109275 on chromosome 1 at Xinxiang and in combined analysis in Pop.1, and phi031–bnlg1617 on chromosome 6 in Pop.2. These results suggested that there were tightly linked QTL controlling grain starch and oil content in these marker intervals.

However, 22 single-trait QTL failed to show significance in multiple-trait analysis, including 6 QTL for starch content at bins 3.07–3.08, 5.02–5.04, 8.06 and 9.03–9.04, and 3 QTL for oil content at bins 6.04 and 10.04–10.05 in Pop.1, and 7 QTL for starch content at bins 3.07–3.08, 5.03/5.03–5.05, 9.03/9.05 and 10.05–10.06, and 6 QTL for oil content at bins 1.03–1.04/1.05–1.06/1.08, 3.06/3.06–3.08 and 8.06 in Pop.2 (Table 3, Table 6). Those QTL might have effects with opposite directions for both traits. QTL at bins 1.03, 3.05, 8.04-8.05 in Pop.1, and at bin 3.03 in Pop.2, were all detected both in single-trait QTL mapping for starch content and in multiple-trait QTL mapping for both traits. These QTL for starch content might not be influenced by oil content.

## Discussion

### Comparison of QTL Detected for Grain Starch Content in the Two Connected RIL Populations and in Previous Studies

In this study, 20 single-population and 16 joint-population QTL for grain starch content were identified for the two connected RIL populations under four environments and in combined analysis. In single-population analysis, no QTL showed consistency across all cases. QTL at the same bins 5.03 and 9.03 were detected in both populations in 4 and 5 cases, respectively. Three QTL on chromosome 3 were located at different bins in both populations, at bins 3.05 and 3.07–3.08 in Pop.1 and at bins 3.02–3.03 in Pop.2. The QTL on chromosomes 1 and 8 in Pop.1, and the QTL on chromosome 10 in Pop.2 could be considered as population-specific QTL. Joint-population analysis showed that only 2 QTL (qcSTA9-1 and qzSTA5-1) had significant effects in both populations. Ten of the 16 joint-population QTL had significant effects in only one population, which could be considered as rare QTL.

In our previous study, two F_2∶3_ populations developed from the same two crosses as in this study had been used to map QTL for grain starch content [10]. In comparison, QTL linked with the same markers at bins 5.04 (umc1162), 8.04 (bnlg2046) and 8.05 (umc1562) in Pop.1, and at bin 5.03 (umc1389) in Pop.2 were observed across both F_2∶3_ and RIL generations. However, QTL showing generation-specific were observed in both populations, which included QTL on chromosomes 6 and 10 in F_2∶3_, and on chromosomes 1, 3 and 9 in RIL for Pop.1, and on chromosomes 1, 2, 4 and 6 in the F_2∶3_ generation, and on chromosomes 3, 9 and 10 in the RIL generations for Pop.2. Great influences of genetic backgrounds (different populations and different generations derived from the same cross) and environments on QTL detection for grain starch content had been commonly reported in previous research [10], [19], [20]. Since the field experiments for the F_2∶3_ and RIL populations were conducted in different years and at different locations, the discrepancies of QTL detected in the two generations could be brought by different genetic backgrounds, environments and their interactions. For the dramatically smaller percentage of variation explained by each QTL at chromosomes 5 and 8 herein, another reason might be the dominance effects at different levels (partial dominance, dominance, and over-dominance) for most QTL detected in the F_2∶3_ generation. Therefore, it was necessary to conduct QTL mapping using various genetic backgrounds under diverse environments thoroughly. Only such consistent QTL would have broad values in theoretical research and practical breeding.

Simultaneously considering the results in other previous research [5], [9], [13], [19], [21], [37], QTL detected herein at all bin loci except bin 8.06 have been detected in 1–4 previous reports (Table 7). qcSTA2-3-1 located at bins 3.02–3.03 with the highest contribution to phenotypic variation (12.35%) has also been identified by Liu et al. [13] and Wassom et al. [9]. The QTL at bin 5.03 with consistency across populations (Pop.1 and Pop.2), generations (RIL and F_2∶3_) and environments has also been identified by Goldman et al. [5], Zhang et al. [19], Wassom et al. [9] and Wang et al. [10]. The QTL at bins 8.04-8.05 with consistency across generations and environments in Pop.1 has also been identified by Zhang et al. [19], Wassom et al. [9] and Wang et al. [10]. The QTL at bin 9.03 with consistency across populations and environments has also been identified by Goldman et al. [5] and Wassom et al. [9].

**Table 7:** Comparison of QTL for grain starch content detected in the two RIL populations and in previous studies.

Bin locus	Environment^a^	Reference	Flanking marker
1.03	Z, A	5, 9, 19, 13	umc1044-phi109275
3.02–3.03	c	9, 13	umc2259-bnlg1447
3.05	Y	5, 9, 13	phi053-umc1174
3.07–3.08	X	5, 9	umc1399–umc1844
5.02–5.04	X, A	5, 9, 10, 19	bnlg1879-umc1162
5.03	z	5, 9, 10, 19	phi008-umc1389
5.03–5.05	x	5, 9, 10, 19	umc1389-umc1722
8.03–8.04	C	10	umc2075-bnlg2046
8.04–8.05	X,Y	9, 10, 19	bnlg2046-umc1562
8.06	Z		umc1149-umc1960
9.03	c, z, a	9	phi065-umc1267
9.03–9.04	C,X	5, 9	umc1688-umc1771
9.05	x	5	umc1657-umc1494
10.05–1.06	a	10	umc1506-bnlg2190

a C, X, Z, Y and A present QTL detected at Xuchang, Xinxiang, Zhengzhou, Yinchuan and in combined analysis in Pop.1, respectively.

c, x, z, y and a present QTL detected at Xuchang, Xinxiang, Zhengzhou, Yinchuan and in combined analysis in Pop.2, respectively.

The biosynthesis process of starch is very complex and is regulated by many genes. QTL co-located with genes encoding for related functions could be considered as candidate genes [40], [41]. ADP-Glc pyrophosphorylase, starch synthase (granule-bound starch synthase and soluble starch synthases), starch branching enzyme and starch de-branching enzyme are four kinds of important enzymes in starch synthesis and accumulation [42], [43], [44]. According to the public genetic linkage map (www.maizegdb.org), several related functional genes were located in the marker intervals of QTL on chromosomes 1, 3, 5 and 8, such as collapsed kernel3 (*p3*), defective kernel1(*dek1*), *dek32*, pyruvate decarboxylase3 (*pdc3*), soft endosperm3(*sen3*), NADH ubiquinone oxidoreductase1(*nad1*) on chromosome 1, beta-D-glucosidase (*glu1*), NADP malic enzyme3 (*me3*), 6-phosphogluconate dehydrogenase (*pdh1*), and *sen1* on chromosome 3, acetolactate synthase 2 (*als2*), *dek27* and sucrose export defective1 (*sxd1*) on chromosome 5, and sugar transport 1 (*stp1*), starch branching enzyme 3 (*sbe3*), and sucrose phosphate synthase1 (*sps1*) on chromosome 8. Further studies should be concentrated on the chromosome intervals of QTL at bins 3.02–3.03, 3.05, 5.03, 8.04–8.05 and 9.03 in near isogenic line (NIL) construction, fine-mapping, and marker-assisted selection (MAS) in starch improvement. In fact, three QTL (qxSTA1-8-1, qySTA1-3-1 and qySTA1-8-1) in the single-population analysis and eight QTL (qcSTA8-1, qcSTA9-1, qxSTA1-1, qxSTA9-1, qzSTA8-1, qaSTA1-1, qaSTA8-1 and qaSTA9-1) in the joint-population analysis at bins 1.03, 1.04–1.05, 3.05, 8.04–8.05, 9.03, and 9.05 could be considered as being fine-mapped. MAS has been used to select their NILs in our present research.

### Comparison of QTL Detected for Starch and Oil Content and their Correlations

In maize kernel, 98% of starch is stored in endosperm and 85% of oil is located in embryo [38]. Selection for oil increase in kernel was accompanied by decrease in kernel weight in IHO [9], [12], a synthetic population [39], and normal corn germplasm [45]. Negative relationship between grain oil content and starch content was commonly reported in research using Illinois strains [4,9,46], BHO [19], ASK high-oil [10], and popcorn germplasm [13]. In the present study, significant negative phenotypic and genotypic correlations between grain starch and oil content were observed for the two RIL populations under each environment and in combined analysis. Comparing with the single-trait QTL detected for starch and oil content, QTL for both traits were all detected on chromosomes 1, 3 and 8 in Pop.1, and on chromosome 3 in Pop.2. Only the QTL at bin 1.03 for both traits was located in the same marker interval (umc1044–phi109275), and with the favorable alleles contributed by the high-oil parent GY220. Specific QTL were observed for grain oil content on chromosome 6 and for grain starch content on chromosome 9. However, tightly linked and/or pleiotropic QTL for both traits were reflected in multiple-trait analysis at bins 1.03, 3.05, 4.05–4.06, 5.05–5.06 and 5.06–5.07 in Pop.1, and at bins 1.05–1.06, 6.03–6.04, 6.04–6.05 and 8.03 in Pop.2. In addition, most single-trait QTL for oil and starch content failed to be detected in multiple-trait analysis. Similar results have been observed by Berke and Rocheford [15], Zhang et al. [19] and Wang et al. [10]. Obviously, the relationship of grain starch content and oil content was very complicated [10], [18]. This reflected that tightly linked and/or pleiotropic QTL with opposite effects might exist at those chromosome regions. It was not easy to improve grain starch and oil content simultaneously.

However, QTL for grain starch and oil content were all detected with all the favorable alleles contributed by the high-oil parent GY220 at bins 1.03 and 8.03–8.04. There might exist tightly linked and/or pleiotropic QTL with the same direction effect for both traits. QTL located at bin 6.03–6.04 for grain oil content might increase grain oil content without reduction in grain starch content. In fact, the high-oil QTL (*qHO6*) cloned by Zheng et al. [20] could increase grain oil content with no effect on seed weight, which was located at bin 6.04. Some candidate genes encoding the enzymes involved in the synthesis and modification pathways of grain oil were located in the marker intervals of QTL on chromosomes 1 and 6, such as fatty acid desaturase8 (*fad8*) and oleic acid content1 (*olc1*) on chromosome 1, and linoleic-acid content (*ln1*) on chromosome 6. Zheng et al. [20] considered that *ln1*and *qHO6* were likely the same genes. Further research should be conducted to prove the effect of these QTL in the simultaneous improvement of grain starch and oil content in maize. Certainly, what is the real situation in our populations should be proved in further research.
